# Familial Syndromes Coupling with Small Renal Masses

**DOI:** 10.1155/2008/413505

**Published:** 2008-08-04

**Authors:** Jorge Hidalgo, Gilberto Chéchile

**Affiliations:** Department of Urology, Instituto Medico Tecnológico, Barcelona 08024, Spain

## Abstract

During the past two decades, several new hereditary renal cancers have been discovered but are not yet widely known. Hereditary renal cancer syndromes can lead to multiple bilateral kidney tumors that occur at a younger age than that at which the nonhereditary renal cancers occur. The aim of our work is to review the features of hereditary renal cancers, the basic principles of genetic relevant to these syndromes, and the various histopathologic features of renal cancer. In addition, we will describe the known familial syndromes associated with small renal masses.

## 1. INTRODUCTION

The incidence of renal cell carcinoma is increasing. This disease affects approximately 150 000 people annually worldwide, causing nearly 78000 deaths [1]. Of these cases, approximately 4% are thought to be associated with autosomal dominant hereditary cancer syndromes [2].

Hereditary renal cancer differs from sporadic renal cancer in several important respects. A hallmark of hereditary renal cancer is that it is often multiple and bilateral.

These distinct forms of inherited epithelial kidney cancer include von Hippel-Lindau disease (VHC), hereditary papillary renal carcinoma (HPRC), Birt-Hogg-Dubé syndrome (BHD), hereditary leiomyomatosis renal cell carcinoma (HLRCC) and renal carcinoma associated with hereditary paraganglioma [2]. The genes for each of these disorders have been identified by positional cloning including the VHL gene, the MET proto-oncogene, the BHD gene, the FH gene, and the SDHB gene [2]. Recently, familial renal carcinoma (FRC) has been described. Families with multiple members with renal carcinoma who do not have one of the known inherited forms of renal carcinoma are considered to have FRC. FRC is currently a diagnosis of exclusion [3].

A small percentage of renal cell carcinomas (RCCs), which are subclassified by histology into clear cell (75% of cases), papillary (10–15%), and chromophobe (5%) RCCs, and renal oncocytoma (3%–5%), are due to inherited cancer syndromes [4]. Each inherited cancer syndrome, such as VHL, HPRC and hereditary leiomyomatosis and renal cell carcinoma (HLRCC), is characterized by the development of specific histologic types of renal cancer [2]. For example, affected members of families with VHL syndrome frequently develop clear cell RCCs, whereas patients with HPRC are predisposed to develop type-1 papillary renal carcinomas [5]. Patients with HLRCC, by contrast, develop aggressive papillary type-2 renal carcinomas [6] (see Table 1).

The widespread use of body imaging in recent years has led to a significant increase in the incidence of renal cell carcinoma (RCC). A distinction between benign and malignant small renal masses cannot be made based on radiographic data alone and percutaneous renal mass biopsy is still controversial [7]. Clinicians therefore, when confronted with small renal masses, must carefully weight the risks and benefits of surgical removal [8].

Herein, we will review the features of hereditary renal cancers, the basic principles of genetic relevant to these syndromes, and the various histopathologic features of renal cancer. In addition, we will describe the known familial syndromes associated with small renal masses.

## 2. VON HIPPEL-LINDAU DISEASE

Von Hippel-Lindau (VHL) disease is an autosomal dominant syndrome that affects multiple organ systems. Extrarenal manifestations of VHL include central nervous system hemangioblastomas, endolymphatic sac tumors, retinal angiomas, pheocromocytomas, and pancreatic cysts and tumors [9].

Renal cancer occurs in 25–45% of patients with VHL; if cystic lesions are included in this estimate, the incidence increases to over 60% [10]. As renal tumors in VHL tend to be multifocal and bilateral and unaffected renal tissue is at risk for developing additional tumors [11], management of these patients is a challenge. The primary goal of managing patients with VHL is prevention of metastatic disease [9]. However, the capacity for CT to detect solid renal masses at an earlier stage increases the importance of secondary goals, such as preservation of renal function and maximization of quality of life, by minimizing the number of surgical procedures that patients must undergo [10].

In VHL, patients inherit a germline mutation of the VHL gene on chromosome 3p25 [12]. The VHL gene encodes pVHL, which is part of a complex (including elongin B/C and CUL2) that targets the *α*-subunit of hypoxia-inducible factors 1 and 2 (HIF1*α* and HIF2*α*) for ubiquitin-mediated proteasomal degradation. If the second copy of the VHL gene in a patient is inactivated, HIF1*α* and HIF2*α* accumulate. This leads to an increased transcription of genes that encode downstream substrates of HIF1*α* and HIF2*α*, such as vascular endothelial growth factor, platelet-derived growth factor and transforming growth factor-*α* (TGF-*α*). These molecules are thought to be important in VHL tumorigenesis [13].

In the pre-CT era, strategies for managing VHL renal tumors were often limited to watchful waiting or bilateral nephrectomy with renal replacement therapy (dyalisis or renal transplantation). The high historical rate of metastasis (13–42%) [14], despite the generally low grade of VHL renal tumors, makes watchful waiting an unappealing strategy. Some researchers have advocated bilateral nephrectomy as a means of removing all renal tissue at risk for tumor development [15].

Performing bilateral nephrectomy necessitates renal replacement therapy. Goldfarb et al. compared 32 patients with VHL who underwent bilateral nephrectomy and subsequent renal transplantation with a matched cohort of renal transplant recipients without VHL [16].

No significant differences in graft survival or renal function were observed between the two groups. Five deaths ocurred in both groups; three in the VHL group were due to metastatic disease. Five-year survival was 65%. The authors concluded that renal transplantation was an effective form of renal replacement therapy for VHL patients with limited risk of cancer recurrence [16].

Nephron-sparing surgery (NSS) has the potential to preserve renal function while maintaining oncology efficacy for appropiately selected patients. Favorable results were achieved in several cohorts of VHL patients undergoing NSS [9].

Factors associated with successful NSS outcomes were small tumor size and low tumor grade; larger tumors (>5 cm) had higher local recurrence and metastatic rates [17].

Even though many VHL patients are young and otherwise healthy, the morbidity of hemodialysis and immunosuppression is significant; five-year survival rates for a cohort of patients demographically similar to VHL patients were 71% on hemodialysis and 86% following renal transplantation [9].

Although salvage partial nephrectomy carries a high rate of perioperative morbidity. However, more than three-quarters of operated kidneys can be preserved with only modest decreases in renal function. These patients are able to avoid or postpone the associated morbidity of dialysis, including some patients with solitary kidney. Oncological outcomes are encouraging at intermediate followup with no evidence of detectable metastatic disease [18].

Undergoing frequent surgeries for small renal masses is not an appealing prospect for VHL patients; an observational strategy in combination with NSS is more attractive.

A cohort of patients with VHL and renal masses were observed until the largest tumor in a renal unit was 3 cm in diameter, at which time surgery was recommended. The pattern of recurrence, bilaterality, and number of tumors were not taken into consideration [14]. The rate of metastases increases with increasing tumor size (Table 2).

The 3 cm threshold is not an absolute threshold demarcating development of metastatic disease; rather it is a point at which the risk of metastasis with the potential morbidities of multiple procedures are balanced [9].

Because of the high rate of recurrence of renal tumors in VHL and the difficulties associated with repeated renal surgery, ablative technologies such as cryoablation and radiofrequency ablation (RFA) have been valid alternatives. Ablative procedures have been performed both laparoscopically and percutaneously [9].

The potential role of the heat-shock protein 90 inhibitor 17-allylamino-17-desmethoxy-geldanamycin in VHC is currently being evaluated in patients with small (2-3 cm) presurgical renal lesion. Inhibition of heat shock protein 90, a molecular chaperone of HIF, facilitates proteasomal degradation of HIF [9]. These approaches, which target the abnormal molecular pathways involved in VHL tumorigenesis, represent a potential future approach to treatment of patients with both sporadic and VHL-associated clear-cell kidney cancer.

NSS-bases approaches to management of VHL-associated renal tumors, using a 3 cm tumor size threshold for recommendation of surgery, can provide good cancer control while preserving renal function and minimizing interventions. This type of strategy mandates diligent screening and followup. More experience with minimally invasive techniques is needed before their role in treatment of VHL renal tumors can be defined. Medical therapy with new molecular-targeted agents is a promising potential development in the management of VHL renal tumors [9].

## 3. HEREDITARY PAPILLARY RENAL CARCINOMA

Papillary renal carcinoma (PRC) comprises 10% to 15% of kidney epithelial tumors and it is histologically subdivided into types 1 and 2 [20]. Hereditary papillary renal carcinoma (HPRC) is an uncommon form of inherited kidney cancer characterized by the predisposition to develop bilateral, multifocal renal tumors with type-1 papillary architecture [21]. Tumors show frequent trisomy of chromosome 7 and they appear to arise from independent clonal events. HPRC is associated with a mutation of the A *MET* proto-oncogene at 7q31.3. The gene was originally described in 1984 but was not linked with papillary renal cancer until 1997 [5]. This gene codes for a transmembrane receptor tyrosine kinase. Mutations lead to activation of the MET protein, which is also the receptor for hepatocyte growth factor. The tumors produced in hereditary papillary renal cancer are well differentiated type-1 papillary renal cancers [22].

Hereditary papillary renal tumors are generally hypovascular and enhance only 10–30 HU after intravenous administration of contrast material. This mirrors the experience with sporadic papillary renal cancers, which are also typically hypovascular. Papillary renal cancers can be mistaken for cysts, and one must be careful to obtain accurate attenuation measurements before and after contrast enhancement. Ultrasonography can be particularly misleading with this disorder, because small tumors are often isoechoic [22].

Patients in HPRC have previously been reported to have renal cancer on average in the sixth decade of life [21], later than other inherited renal cancer syndromes such as VHL disease, which often develops in patients in the third and fourth decades of life. Schmidt and colleagues reported on 3 families with HPRC in which, individuals in HPRC are at risk for bilateral, multifocal kidney cancer earlier in life (second decade) [23]. In addition, this report emphasizes that HPRC can be a lethal disease since a number of affected individuals in these families died of metastatic kidney cancer. Type-1 papillary renal carcinoma in patients with HPRC is a malignant tumor that can be lethal if it is not detected and treated early [23].

## 4. BIRT-HOGG-DUBÉ SYNDROME

Birt-Hogg-Dubé syndrome (BHD) is an autosomal dominant cancer syndrome characterized by the development of small dome-shaped papules on the face, neck, and upper trunk (fibrofolliculomas). In addition to these benign hair follicle tumors, BHD confers and increases the risk of renal neoplasia and spontaneous pneumothorax. The gene has been mapped to chromosome 17p11.2 and recently identified, expressing a novel protein called folliculin [24].

Recently, individuals with BHD syndrome were found to have a seven-fold higher risk over the general population of developing kidney neoplasms [25].

Unlike renal tumors in patients with other inherited kidney cancer syndromes, renal tumors from BHD patients exhibit a spectrum of histologic types, including chromophobe (34%), oncocytoma (5%), clear cell (9%), papillary (2%), and an oncocytic hybrid (50%) with features of chromophobe RCC and renal oncocytoma [26]. Germline mutations have been identified in a novel gene, BHD in affected family members [27]. BHD encodes a protein, folliculin, which is named for the hallmark dermatologic lesions found in BHD patients. All germline mutations identified to date are frameshift or nonsense mutations that are predicted to truncate folliculin, including insertions or deletions of a tract of eight cytosines (C8) in exon 11 [27].

Pavlovich et al. [26] reported 130 solid renal tumors resected from 30 patients with BHD in 19 different families. Preoperative CT demonstrated a mean of 5.3 tumors per patient (range 1–28 tumors), the largest tumors averaging 5.7 cm in diameter (±3.4 cm, range 1.2–15 cm). Multiple and bilateral tumors were noted at an early age (mean 50.7 years). The resected tumors consisted predominantly of chromophobe renal cell carcinomas (34%) or of hybrid oncocytic neoplasms that had areas reminiscent of chromophobe renal cell carcinoma and oncocytoma (50%). Twelve clear cell (conventional) renal carcinomas (9%) were diagnosed. The tumors were on average larger (4.7 ± 4.2 cm) than the chromophobe (3.0 ± 2.5 cm) and hybrid tumors (2.2 ± 2.4 cm). Microscopic oncocytosis was found in the renal parenchyma of most patients, including the parenchyma of five patients with evidence of clear cell renal cell carcinoma. These findings suggest that microscopic oncocytic lesions may be precursors of hybrid oncocytic tumors, chromophobe renal cell carcinomas, and perhaps clear cell renal cell carcinomas in patients with BHD syndrome.

The malignant nature of BHD associated renal tumors has not been previously established. BHD associated kidney cancer has the potential to be a lethal disease based on family histories of death from metastatic RCC in several patients with BHD. Pavlovich and colleagues [28] suggest that BHD-associated chromophobe and hybrid oncocytic RCC may be of lesser malignant potential than BHD associated clear cell RCC but these lesions cannot be considered completely benign based on their cytomorphology and the known occasionally malignant behavior of chromophobe tumors. In families in which multiple members are found to have chromophobe or hybrid oncocytic renal carcinomas, BHD should be considered. Efforts are currently underway to determine why some BHD families have kidney cancer and others do not [28].

Urological surgeons who treat patients with BHD should keep in mind the potential for perioperative pneumothorax in these patients. A high percent of patients with BHD is affected with pulmonary cysts (almost 90%) and more than 20% have a history of spontaneous pneumothorax.

To address the mutation status of the BHD gene in tumors from Birt-Hogg-Dubé patients, Vocke and colleagues [29] analized a panel of 77 renal tumors by direct DNA sequence analysis. Tumor samples, as well as matched normal samples, were obtained from 12 affected members of BHD families after renal surgery. BHD patients were often found to have bilateral, multifocal tumors and underwent staged bilateral partial nephrectomies, providing tumor samples for the study. The entire coding region of BHD (exons 4–14) was sequenced in each tumor sample, following polymerase chain reaction (PCR) amplification. Their data showed that the tumors from a given BHD patient have different second hits. These observations strongly suggest that multiple renal tumors from some BHD patients are independent, clonal events, each arising from a separate and unique second mutation in the BHD gene. However, some tumors with mixed histologies shared a common somatic mutation in the distinct histologic regions within each tumor. This finding suggests that in some cases, a somatic second hit precedes histologic diversification within a single tumor. The molecular mechanism that drives these events is unknown. These results document the high frequency and wide spectrum of second mutations, which strongly support a tumor suppressor role of BHD. Inactivation of both copies of BHD ocurred in several histologic types of renal tumors, suggesting that BHD may act at an early stage of renal oncogenesis. Further understanding of the mechanism of BHD-induced tumorigenesis awaits functional studies of the follicullin protein [29].

In addition, BHD is an autosomal dominant hereditary cancer syndrome, in which affected individuals are at risk for cutaneous fibrofolliculomas, pulmonary cysts, spontaneous pneumothorax, and kidney tumors. Almost 30% of affected patients with BHD examined had solid renal tumors. Because of the spectrum of renal tumor histologies found in patients with BHD, their variable natural history, and the risk of recurrent renal tumors in such patients, it is important for urologists to be aware of this syndrome. The current management approach for BHD associated renal tumors is to perform nephron sparing surgery when possible [28].

## 5. HEREDITARY LEIOMYOMATOSIS AND RENAL CELL CANCER

A hereditary form of kidney cancer referred to as hereditary leiomyomatosis and renal cell cancer (HLRCC) has been identified, in which affected family members have cutaneous leiomyomas, uterine fibroids, and/or kidney cancers [6]. The renal malignancies that develop in HLRCC families are often metastatic at presentation and are a significant cause of mortality in these families. Analysis of families with this disorder has identified the responsible gene locus as FH [30]. This gene encodes fumarate hydratase (FH), an enzyme that is part of the mitochondrial Krebs or tricarboxylic acid (TCA) cycle, located at 1q42.2-42.3. The gene is inherited in an autosomal dominant manner. The mechanism by which alterations in FH lead to HLRCC remains to be determined, but it apparently involves increased cellular dependence on glycolysis.

The reason why *FH* alterations are associated with tumor formation in HLRCC families is not entirely clear at this time. It seems intuitively that a cell that lacks functional FH (and hence has a defective TCA cycle) would be at a metabolic disadvantage, particularly with regard to the efficiency of nutrient catabolism. HLRCC is not, however, the only hereditary cancer syndrome associated with a defective enzyme of the Krebs cycle. Germline mutations in the succinate dehydrogenase complex have been identified that predispose to the development of hereditary paragangliomas. Succinate dehydrogenase catalyzes the conversion of succinate to fumarate—the step in the TCA cycle that immediately precedes the reaction catalyzed by FH. Mutations in subunits B, C, and D of the succinate dehydrogenase complex have all been linked to hereditary paraganglioma.

The extrarenal manifestations of HLRCC were described previously [6]. The most frequent manifestation is uterine leiomyomas in affected females (75% to 98%). More than 90% of the women underwent myomectomy or hysterectomy and approximately half had undergone hysterectomy by age of 30 years. Cutaneous leiomyomas are firm, skin-colored to light brown or red papules. They may be segmental and multifocal, and are mainly found on the trunk and extremities. They may be painful. Mean age at onset of cutaneous manifestations is 25 years (range 10 to 47) [31]. The incidence is 36% to 85%.

There are several unique aspects to HLRCC-associated renal tumors that differentiate them from other inherited forms of kidney cancer. Whereas tumors in VHL, HPRC, and BHD twins are often multifocal and involve the 2 kidneys [2], renal tumors in patients with HLRCC may be solitary.

Toro et al. [31] reported 19 patients with hereditary leiomyomatosis and renal cell cancer associated renal tumors. Individual considered affected by HLRCC had greater than 10 skin lesions clinically compatible with leiomyoma and a minimum of 1 lesion histologically confirmed as leiomyoma or tested positive for a germline FH mutation. Patients underwent precontrast and postcontrast CT of the chest, abdomen, and pelvis after informed consent was provided. Renal lesions were considered indeterminate if they were too small (less than 1 cm in diameter) to be accurately classified as solid or cystic. Only lesions 1 cm or greater and enhancing more than 20 HU that were predominantly solid were considered renal tumors.

For HLRCC they did not adhere to the strategy of expectant management for tumors less than 3 cm, as we previously described for other hereditary kidney cancer syndromes, such as VHL, HPRC, or BHD [2, 19, 28].

HLRCC-associated renal tumors appear to represent a significantly more aggressive type of renal cancer than that in patients with VHL, HPRC, or BHD. Even small HLRCC renal tumors are associated with nodal and metastatic disease. In the reported study [32], they often treat patients with VHL, HPRC, and BHD and small (less than 3 cm) tumors expectantly, recommending surgery in many when the largest lesion reached 3 cm. To their knowledge no patients with VHL, HPRC, or BHD who presented with tumors less than 3 cm have had metastatic disease using this clinical management approach [14, 19]. Because of the aggressive nature of the renal cancer in HLRCC in the current study [32] and the potential for small tumors to metastasize, the data suggest that small lesions prospectively identified in patients at risk for HLRCC should not be managed by an expectant, nonsurgical strategy. Experience with nephron sparing surgery in the setting of HLRCC is limited to date and no formal recommendations regarding the most efficacious surgical approach (radical versus partial nephrectomy) for clinically localized renal tumors can be made at this time, nephron-sparing surgery could be potentially as curative as radical nephrectomy as has been demonstrated in nonhereditary forms of RCC.

A family history of renal tumors, especially causing death at a young age, early hysterectomy in women due to symptomatic fibroids, cutaneous leiomyomas, and importantly small tumors with a lymph node or metastatic disease burden out of proportion to tumor size should alert clinicians to the possibility of HLRCC. Renal tumors found in this syndrome, which are frequently described as papillary type II or collecting duct histology, appear to be significantly more aggressive than other forms of hereditary renal cancer. Because of limited experience with screening and treating these patients, optimal management strategies remain to be defined. However, the early experience with HLRCC-associated renal carcinoma suggests that extreme caution is warranted. Observational strategies that are suitable for select patients with small renal masses associated with other hereditary renal cancer syndromes are not appropriate for patients with HLRCC. HLRCC-associated kidney cancer is markedly different from kidney cancer associated with other hereditary cancer syndromes, such as VHL, HPRC, and BHD. These patients should be evaluated and treated cautiously [32].

## 6. RENAL CARCINOMA ASSOCIATED WITH HEREDITARY PARAGANGLIOMA

Germline mutations of the genes encoding succinate dehydrogenase subunits B (SDHB) and D (SDHD) predispose to paraganglioma syndromes type-4 (PGL-4) and type-1 (PGL-1), respectively. In both syndromes, pheochromocytomas as well as head and neck paragangliomas occur; however, details for individual risks and other clinical characteristics are unknown.

The paraganglioma syndromes have been relatively newly delineated as unique entities. Although paraganglioma has been clinically recognized for more than 40 years, only in the last 4 years they have been classified based on molecular genetics: SDHD mutations predispose to PGL-1, mutations in an unidentified gene on chromosome 11 to PGL-2, SDHC mutations to PGL-3, and SDHB mutations to PGL-4. In Neumann et al. report [33], consistent with the apparently aggressive nature of SDHB dysfunction, 5 mutation carriers in their study were also found to have extra paraganglial malignancies (e.g., renal cell carcinoma and thyroid papillary carcinoma). Kidney carcinomas are considered oncocytic tumors (replete with mitochondria) and thus, the involvement of a mitochondrial complex II gene in kidney carcinogenesis may be explained. The apparently more aggressive nature of the tumors in SDHB mutation carriers may be postulated to be a consequence of the prevention of assembly of the catalytic complex that normally comprises SDHA and SDHB, thus leaving only complexes of the structural SDHC and SDHD moieties.

Mutations in other genes can be causes of hereditary renal carcinoma. These genes include hepatocyte nuclear factor 1 *α* and 1 *β*, the tuberous sclerosis genes and and SDHB [33].

## 7. FAMILIAL RENAL CARCINOMA

There are several interrelated questions when approaching the family with multiple members with renal carcinoma. The first issue is determining whether the family is affected with one of the known inherited forms of renal carcinoma. The diagnosis of one of the known inherited forms of renal carcinoma is based on clinical evaluation and DNA testing. Families with 2 or more members with renal carcinoma who do not have one of the known inherited forms of renal carcinoma are considered to have FRC. Recently, Zbar et al. [3], reported a study in which familial renal carcinoma (FRC) was described and provisionally clasified (Table 3). They evaluated 141 at risk asymptomatic relatives of affected individuals from 50 families with 2 or more members with renal carcinoma. Histology slides of renal tumors from affected family members were reviewed and were not found to be VHL, BHD, HLRCC, or HPRC. At risk, members from renal carcinoma families were screened for occult renal neoplasms by renal ultrasound and computerized tomography. DNA from selected families was tested for germline mutations of known renal carcinoma genes when clinically indicated and constitutional cytogenetic analysis was performed to search for germline chromosome alterations. This collection of renal carcinoma families represents a well-studied population from which families with the 4 well-known causes of inherited renal carcinoma were removed from the study.

Findings suggested that, when confronted with a family with FRC, careful analysis should be performed of the family to search for known causes of inherited renal carcinoma (Table 4). The manifestations of hereditary leiomyoma renal carcinoma may be particularly difficult to identify. The most likely cause of aggressive, early onset FRC was hereditary leiomyoma renal cell carcinoma. In general, bilateral multiple renal carcinomas in more than 1 family member are highly suggestive of an autosomal dominant form of renal carcinoma. If there is a suggestion of hereditary renal cancer, appropriate biopsies and scans should be performed and DNA mutation studies should be performed to confirm the diagnosis.

## 8. CONCLUSIONS

Small renal masses in the case of renal cancer syndromes must be studied from a particular point of view because hereditary renal cancers can lead to multiple and/or bilateral kidney tumors. The primary goal of managing patients with familial renal syndromes is prevention of metastatic disease. Nephron sparing surgery has the potential to preserve renal function while maintaining oncology efficacy for selected patients. In some syndromes, it is appropiate to develop a watchful waiting attitude, 3 cm size tumor seems to be the threshold for renal surgery. Due to the aggressive form of renal carcinoma in the case HLRCC, initial surgical approach is recommended.

## Figures and Tables

**Table 1:** Characteristics of autosomal dominant (AD) forms of kidney cancer (adapted from Zbar et al. [3]).

Disease	Gene	Renal Tumor histology
VHL	VHL	Clear cell Ca
HPRC	MET	Papillary type 1
HLRCC	FH	Renal cell Ca, HLRC type
BHD	BHD	Chromophobe/hybrid oncocytic neoplasm/clear cell Ca

**Table 2:** Comparision of tumor size and metastaes (adapted from Duffey et al. [19]).

Tumor Size (cm)	Number of metastases	Number of patients	Percentage of patients with metastases
≤3.0	0	108	0
3.1–4.1	1	27	4
4.1–5.5	4	19	21
5.6–10.0	10	20	50
≥10.1	5	7	71

**Table 3:** Clinical and pathological subtypes of familial renal carcinoma (adapted from Zbar et al. [3]).

(1) Single clear cell renal carcinomas
(2) Bilateral multiple clear cell renal carcinomas, without VHL
(3) Single clear cell renal carcinomas and renal oncocytomas*
(4) Single clear cell renal carcinomas and papillary renal carcinomas**
(5) Single and multiple renal oncocytomas without the other clinical features of BHD syndrome
(6) Single or multiple bilaterally renal carcinomas but not HPRC or HLRCC
(7) Other
*Families with some members affected with single clear cell renal carcinomas and other members affected with single renal oncocytomas
**Families with one member affected with single clear cell renal carcinoma and another member affected with single clear cell renal carcinoma and another member affected with papillary renal carcinoma

**Table 4:** FRC versus autosomal dominant (AD) forms of kidney cancer. (adapted from Zbar et al. [3]).

Disease	Inheritance mode	Age at onset	Tumor multiciplicity	Histology
FRC	Complex	Late	Single	varied
VHL	AD	Adolescence	Bilat, multiple	Clear cell
HPRC	AD	40–49 years	Bilat, multiple	Papillary type 1
BHD	AD	30–39 years	Bilat, multiple	Chromophobe/hybrid oncocytic
HLRCC	AD	10–20 years	Single or multiple	Renal cell Ca, HLRCC type
